# Commentary: Baseline systemic immune-inflammation index and a novel FLIPI-SII model predict POD24 and long-term survival in grade 1–3a follicular lymphoma

**DOI:** 10.3389/fimmu.2026.1948746

**Published:** 2026-08-26

**Authors:** Guilan Cheng, Kewei Yang, Dan He, Yue Liu, Hongmiao Li, Liangying Zhang, Qingxian Jia, Yuanping Yin, Zhihan Zhao

**Affiliations:** 1 Liaoning Agricultural Vocational and Technical College, Yingkou, China; 2 The Second Affiliated Hospital of Liaoning University of Traditional Chinese Medicine, Shenyang, China

**Keywords:** FLIPI-SII, follicular lymphoma, POD24, prognostic model, risk stratification, systemic immune-inflammation index

## Defining the current role of FLIPI-SII: a prognostic stratification tool rather than a treatment-selection model

Yu et al. recently evaluated the prognostic relevance of the baseline systemic immune-inflammation index (SII) in patients with grade 1–3a follicular lymphoma (FL). They developed a six-item FLIPI-SII model by incorporating SII into the conventional Follicular Lymphoma International Prognostic Index (FLIPI) (1). This study addresses an important clinical need because disease progression within 24 months (POD24) identifies a subgroup of patients with FL who experience particularly unfavorable outcomes (2). The routine availability of SII, together with internal and multicenter external validation, makes FLIPI-SII an attractive and pragmatic approach for risk stratification.

The key interpretative question, however, is how far such prognostic information can be extended toward treatment-related decision-making.

Yu et al. primarily describe SII as an independent prognostic biomarker and FLIPI-SII as a prognostic model. At the same time, the study evaluates SII in patients receiving rituximab-based immunochemotherapy and discusses potential links between systemic immune-inflammatory status, rituximab treatment resistance, and personalized immunotherapy strategies (1). These treatment-oriented interpretations make it important to distinguish prognostic stratification from prediction of differential treatment benefit.

In our view, the current evidence supports FLIPI-SII most strongly as a pragmatic prognostic stratification tool, whereas its role as a treatment-selection biomarker remains unproven.

## Distinguishing prognostic information from treatment-effect prediction

High baseline SII remained independently associated with inferior overall survival (OS) and progression-free survival (PFS), and this association persisted among patients receiving rituximab-based immunochemotherapy (1). These findings indicate that systemic immune-inflammatory status provides clinically meaningful prognostic information in the immunochemotherapy era.

However, outcome prediction among treated patients should be distinguished from prediction of differential treatment benefit. A predictive biomarker identifies whether the relative effect of a specific therapy differs according to biomarker status and is generally evaluated through a treatment-by-biomarker interaction within an appropriate comparative setting (3).

In the cohort studied by Yu et al., most patients receiving frontline immunochemotherapy were treated with R-CHOP, and no sufficiently balanced comparator group was available to determine whether the relative benefit of rituximab differed according to SII status (1). Therefore, the observation that patients with high SII experienced poorer outcomes despite rituximab-based treatment demonstrates persistent prognostic risk but does not establish that these patients derive less benefit from rituximab or would preferentially benefit from alternative therapeutic strategies.

This distinction does not diminish the clinical relevance of FLIPI-SII. Prognostic models can support risk communication, follow-up planning, and clinical-trial enrichment without necessarily identifying the optimal treatment strategy for individual patients. Similar to other modern prognostic models such as FLIPI24, FLIPI-SII should currently be interpreted as an extension of risk stratification rather than a treatment-selection algorithm (4).

## Interpreting FLIPI-SII within its prognostic framework

POD24 represents a clinically meaningful but methodologically sensitive endpoint because its classification depends on adequate assessment of progression status during the first 24 months after treatment initiation (2). Yu et al. excluded patients with less than six months of follow-up and defined POD24 according to confirmed progression within 24 months after frontline treatment (1). Clarification of the number of patients with evaluable 24-month progression status and the handling of patients censored before this time point would further facilitate interpretation and reproducibility of the reported association between FLIPI-SII and early progression risk.

Multicenter external validation supported the model’s potential generalizability (1). Additional evaluation of calibration across independent populations would provide complementary evidence regarding whether predicted risks remain consistent across different clinical settings, because calibration is an important component of prognostic model evaluation (5).

The comparison between FLIPI-SII and FLIPI24 also warrants cautious interpretation. Yu et al. reported no statistically significant difference in PFS discrimination between the two models and described their performance as statistically equivalent or non-inferior (1). However, the absence of a statistically significant difference in a superiority comparison does not formally establish equivalence or non-inferiority. The findings may therefore be more precisely interpreted as showing no statistically detectable difference in model discrimination within the analyzed cohort.

More broadly, improved prediction performance does not automatically demonstrate clinical benefit. The reported reclassification analyses suggest that incorporating SII may provide additional prognostic information (1), but whether FLIPI-SII-guided risk classification improves clinical management remains uncertain (6). At present, the available evidence supports risk stratification rather than an FLIPI-SII-directed treatment strategy.

## The future scope of FLIPI-SII in contemporary follicular lymphoma management

The treatment context of the original study further defines the current applicability of FLIPI-SII. The development cohort largely reflected chemotherapy-based anti-CD20 treatment, whereas the external validation cohort consisted entirely of R-CHOP-treated patients (1). Accordingly, the current evidence most directly supports the prognostic application of FLIPI-SII in patients receiving chemotherapy-based immunochemotherapy.

The therapeutic landscape of FL continues to evolve, with increasing use of bendamustine-based regimens, chemotherapy-free approaches, bispecific antibodies, cellular therapies, and other immune-directed strategies (7). Validation across contemporary treatment settings will therefore be important to determine whether baseline immune-inflammatory status maintains consistent prognostic value when therapeutic mechanisms differ substantially.

Such studies may also clarify whether FLIPI-SII can eventually contribute to treatment-informed decision-making. Establishing such a role, however, would require evidence beyond prognostic association, including comparative treatment analyses capable of determining whether treatment effects differ according to FLIPI-SII-defined risk.

## Conclusion

Yu et al. provide important evidence that baseline SII adds host immune-inflammatory information to conventional clinical risk stratification in grade 1–3a FL. The simplicity of FLIPI-SII, the routine availability of its components, and its multicenter validation make it a promising prognostic model. The central issue is not whether FLIPI-SII has clinical value, but how its clinical role should currently be defined. Available evidence supports FLIPI-SII as a pragmatic prognostic stratification tool that identifies patients at increased risk of adverse outcomes. In contrast, prediction of differential treatment benefit requires a distinct evidentiary framework and remains unproven. Therefore, FLIPI-SII should currently be regarded as a practical prognostic stratification tool rather than a treatment-selection model. Future validation across contemporary treatment settings and comparative biomarker analyses will help determine whether its clinical role can ultimately extend toward treatment-informed decision-making.
